# Introducing 3D printed models of the upper urinary tract for high-fidelity simulation of retrograde intrarenal surgery

**DOI:** 10.1186/s41205-021-00105-9

**Published:** 2021-06-07

**Authors:** Luca Orecchia, Diego Manfrin, Stefano Germani, Dario Del Fabbro, Anastasios D. Asimakopoulos, Enrico Finazzi Agrò, Roberto Miano

**Affiliations:** 1 Urology Unit, Policlinico Tor Vergata Foundation, Viale Oxford 81, 00133 Rome, Italy; 2 Medics3D S.r.L, Moncalieri, Italy; 3 Division of Urology, San Giovanni Bosco Hospital, Turin, Italy; 4 Division of Urology, Department of Surgical Sciences, University of Rome Tor Vergata, Rome, Italy

**Keywords:** Urology, Ureteroscopy, Lithotripsy, RIRS, 3D, Anatomy, Kidney, Simulation, Training

## Abstract

**Purpose:**

Training in retrograde intrarenal surgery for the treatment of renal stone disease is a challenging task due to the unique complexity of the procedure. This study introduces a series of 3D printed models of upper urinary tract and stones designed to improve the training process.

**Methods:**

Six different models of upper urinary tract were algorithmically isolated, digitally optimized and 3D printed from real-life cases. Soft and hard stones in different sizes were produced from 3D printed moulds. The models were fitted onto a commercially available part-task trainer and tested for retrograde intrarenal surgery.

**Results:**

Each step of the procedure was simulated with extraordinary resemblance to real-life cases. The unique anatomical intricacy of each model and type of stones allowed us to reproduce surgeries of increasing difficulty. As the case-load required to achieve proficiency in retrograde intrarenal surgery is high, benchtop simulation could be integrated in training programs to reach good outcomes and low complication rates faster. Our models match incredible anatomical resemblance with low production cost and high reusability. Validation studies and objective skills assessment during simulations would allow comparison with other available benchtop trainers and the design of stepwise training programs.

**Conclusions:**

3D printing is gaining a significant importance in surgical training. Our 3D printed models of the upper urinary tract might represent a risk-free training option to hasten the achievement of proficiency in endourology.

## Background

Renal stone disease represents a significant burden for healthcare systems worldwide. In view of its multifactorial aetiology, its prevalence ranges from 1 to 20 % reaching > 10 % in countries with a high standard of living [1]. It is the second most expensive urological disease, with accruing costs associated with urgent care for symptomatic stones and novel technologies used for treatment [2].

Operative approaches to renal stones are rapidly evolving, intending to reduce the invasiveness of the procedure while ensuring high stone free rates. The current treatment options include extracorporeal shockwave lithotripsy alongside endoscopic approaches, either percutaneous or transurethral (Retrograde Intrarenal Surgery, RIRS).

RIRS is a fully endoscopic and minimally invasive type of surgery. Performing RIRS presents unique challenges due the narrowness of the operating field, the high anatomical variety within each collecting system, the complexity of the operating manoeuvres, the fragility of the dedicated instruments and potentially severe associated complications [3, 4].

Hence, reaching adequate proficiency with RIRS might represent a demanding task for the urologist in training both for the intrinsic technical challenges and the difficulty to achieve an adequate case-load during the residency years [5]. Concurrently, due to the necessity to guarantee patient safety during the entire procedure, having novices beginning their training directly on real-life cases might not represent the most convenient way of approaching RIRS. Therefore, this has led to a growing interest in risk-free alternatives to the traditional surgical apprenticeship model and to the development of several endourology simulation programs using: animal/cadaveric models, virtual trainers and benchtop models [6].

Out of the wide array of technologies applied to simulation, 3D printing had a significant impact in urology thanks to the incredible anatomical accuracy of the printed models and the low costs associated. It has been successfully used to improve patient education, pre-operative planning and simulation-based training [7–9].

To improve the modular approach to teaching RIRS practised in the operating theatre at our institution and possibly decouple it from its strict dependency from the surgical case-load, this study reports on the production and experimentation with a series of completely 3D printed models of upper urinary tracts and stones, devised as a benchtop simulation-based addition to traditional training.

## Methods

### Development

Anonymised Digital Imaging and Communication in Medicine (DICOM) files from Computerised Tomography Urogram (CTU) scans were collected by expert endourologists from real renal stone cases. A total of six CTUs were selected after reviewing each individual anatomy in terms of type and intricacy of the pelvicalyceal system, thus allowing to plan for training models of different complexity. The DICOM files were sent to a bioengineering company (Medics srl, Moncalieri, Italy) for data extraction and modelling. A Region of Interest (ROI) comprising the selected kidney and proximal ureter was designed using the software Mimics 23.0 (Materialise NV, Leuven, Belgium). DICOM files were subsequently segmented using automatic and semi-automatic algorithms for the isolation of the voxels corresponding to the anatomical detail of interest by the identification of variations in Hounsfield Units (HU) in the different CTU phases for each voxel in the selected ROI.

**Fig. 1:**
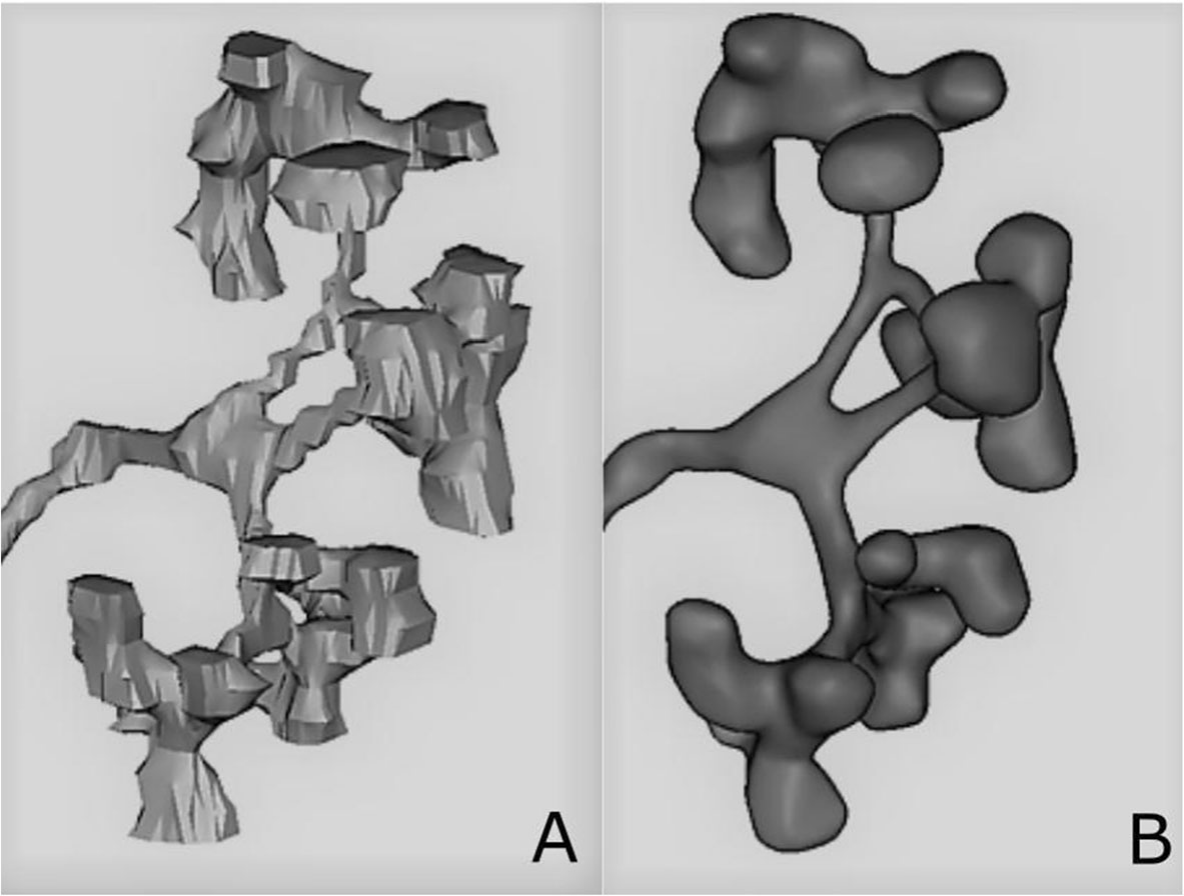
**A** 3D triangulated mesh after interpolation. **B** Post-processed model

The result of the automatic process was then refined and validated by a biomedical engineer subject matter expert. The voxel volume was subsequently converted and interpolated in a 3D triangulated mesh (Fig. 1A). The resulting model was then exported and finalized in 3-matic 15.0 (Materialise NV, Leuven, Belgium). The process included manual noise reduction and smoothing of parts included in the mesh due to approximation in HU readings of the CTU scan volume. The post-processed model was then imported back in Mimics and correspondence with the CTU scan ROI was confirmed (Fig. 1B).

**Fig. 2:**
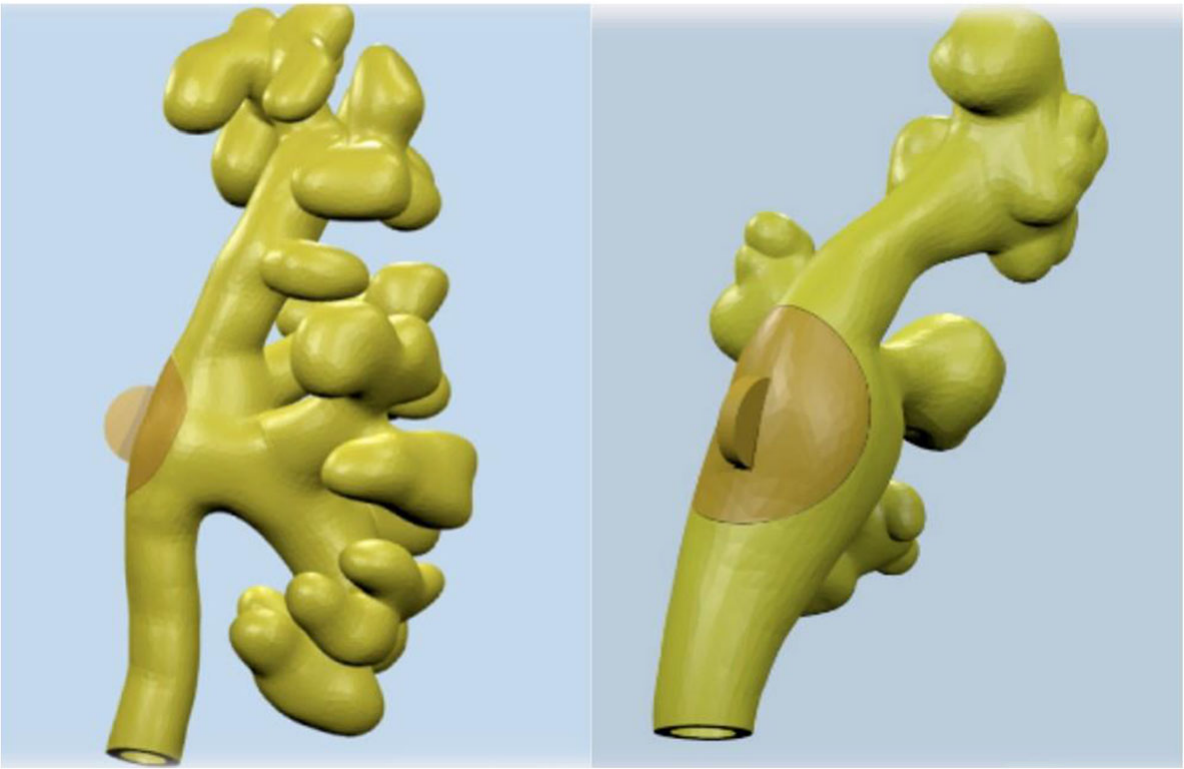
Finalised 3D model in two projections, the elliptical hatch for stone insertion is visible on the side of the renal pelvis

As the original post-processed geometry was solid and completely closed, an external hollowing of 2.5mm was applied to the model in order to allow for real-life endoscopic navigation. Lastly, an expert 3D modeler performed project optimization on the 3D mesh and designed an elliptical hatch with a dedicated press fit closure system for stone insertion in the kidney pelvis during the simulation (Fig. 2). The finalised mesh was then exported in stereolithography (.stl) format for the printing phase.

A A2v4 (3ntr, Oleggio, Italy) or a Ultimaker S5 (Ultimaker, Utrecht, Netherlands) 3D printer was used for dual extrusion printing. A white thermoplastic polyurethane 2.85 mm filament was used for the pelvicalyceal system in order to grant some flexibility during model setup, while a water soluble polyvinyl alcohol filament was used for the inner support scaffold used during the printing phase. The pelvis hatch closure was printed separately from the main structure. After printing the models were submerged in warm water in order to allow the dissolution and detachment of the scaffold system.

**Fig. 3:**
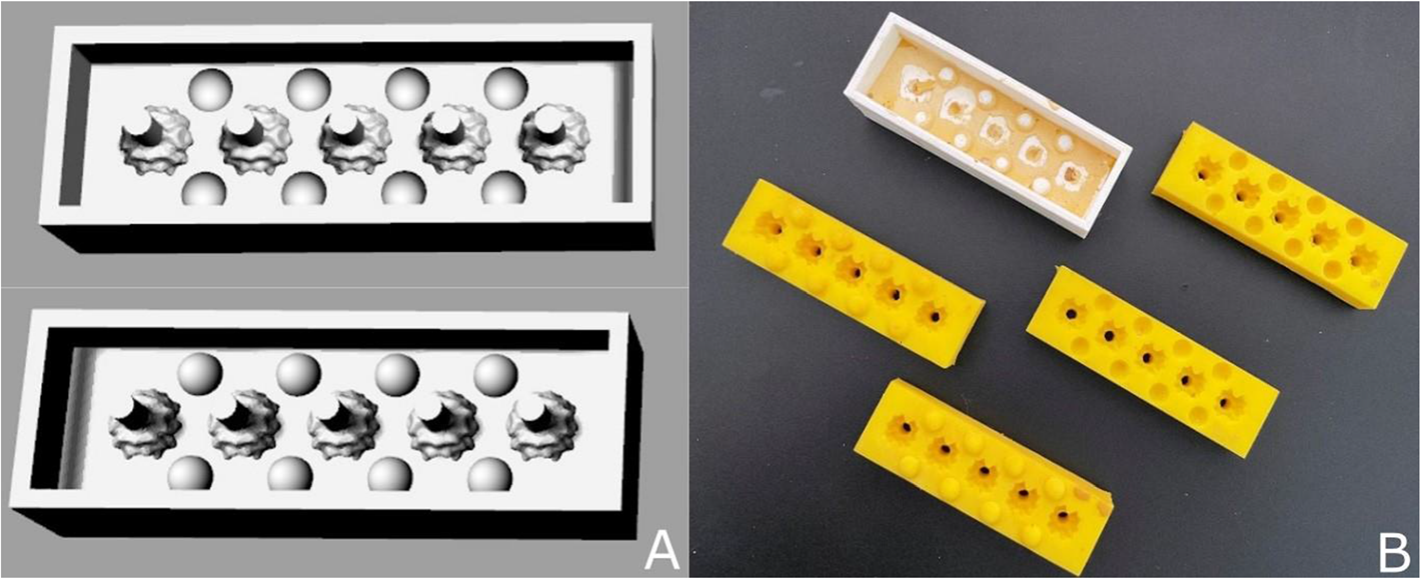
**A** Two halves of the 3D model of the moulds, respectively with embossed and engraved registration spheres. **B** Silicone moulds (yellow) and jig (white)

Training stones were produced after several laser lithotripsy simulations with different compounds which led to the selection of two suspensions of water and chalk in fixed proportions in order to produce soft and hard stones resembling real-life conditions in terms of resistance to lithotripsy. Soft stones were obtained by mixing in a 1:1 chalk to water ratio by weight, while hard stones required a 1.5:1 ratio. The differences in stone hardness were devised to allow the use of different lithotripsy techniques during the simulation (dusting vs. regular/popcorn fragmentation). Casts allowing production of spheroids of different radius were therefore software modelled in negative, 3D printed and used to obtain moulds using bicomponent 1:1 pouring silicone rubber GLS Pro 20 (Prochima, Colli al Metauro, Italy) (Fig. 3). The water and chalk mixtures were then poured through a dedicated channel in the mould and then dried in a food dehydrator to obtain the training stones. Production costs were monitored during the entire process.

### Testing

**Fig. 4:**
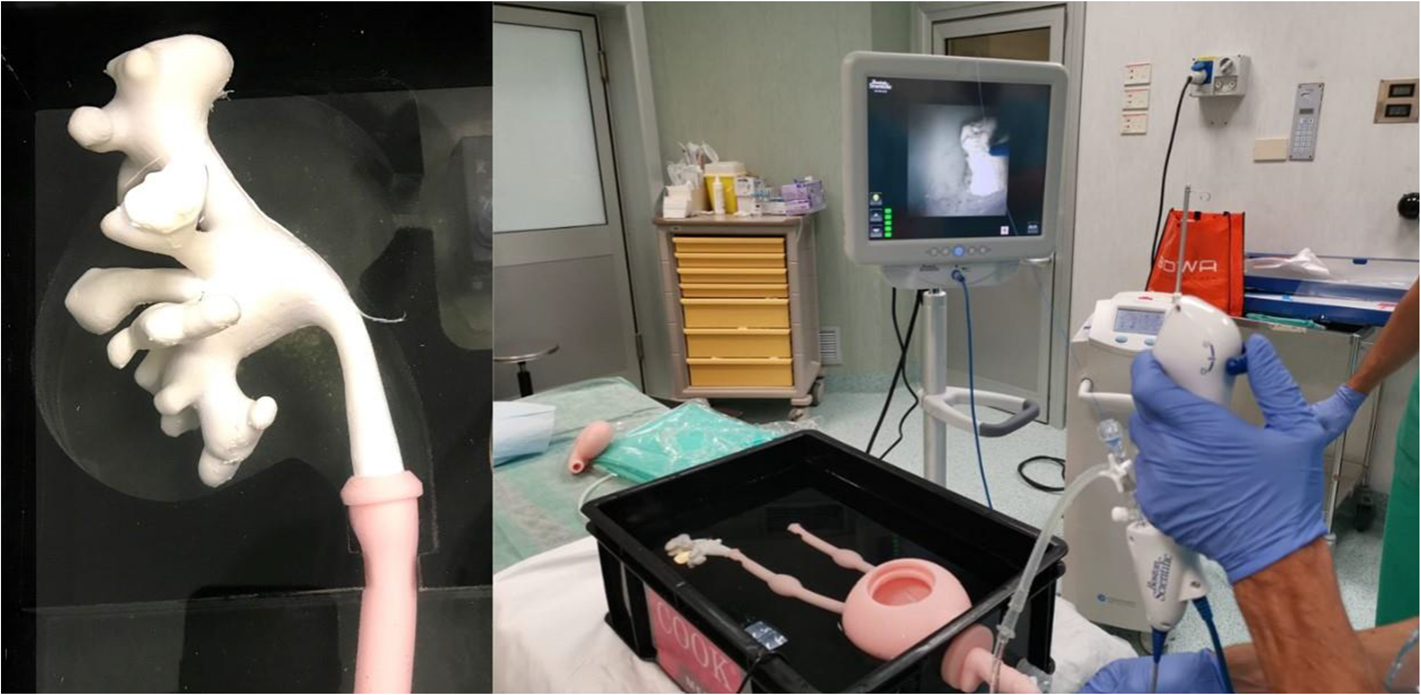
Complete setting in the operating theatre during the simulation

After completion of the development process, the 3D printed models were tested using a commercially available version of Cook Medical ureterorenoscopy (URS) part-task trainer (Cook Medical, Bloomington, IN, USA), consisting of a water filled box containing a bladder-dual ureter system connected to a silicone gel model of a penis for realistic instrument insertion and upper urinary tract drainage [10]. The 3D printed models were press fitted into the proximal extremity of the ureters thus obtaining a complete reproduction of the urinary tract. A 9.5Fr/11.5Fr 35 cm Flexor ureteral access sheath (Cook Medical) was inserted in the trainer over a 0.035 inch HiWire nitinol hydrophilic guidewire (Cook Medical). The models were examined using both a Flex-X2 flexible ureterorenoscope (Karl Storz SE & CO., Tuttlingen, Germany) and a LithoVue single use flexible ureterorenoscope (Boston Scientific, Marlborough, MA, USA). External examination and endoscopic navigation were independently performed by two expert endourologists (RM, SG) in order to rank the anatomical complexity and the level of challenge of URS in each model with a three-tiered complexity score (low, medium, high), results were compared and possible disagreement was resolved through open discussion. One model was extensively tested in an operating theatre to simulate several complete RIRS using an Odyssey 30 Holmium Laser System (Convergent Laser Technologies, Alameda, CA, USA) equipped with a 365 μm Cook Medical Holmium Laser Fibre, a NGage Nitinol Stone Extractor (Cook Medical) was utilised for stone relocation and fragment extraction (Fig. 4). Fluoroscopy was performed during testing using a C-Arm. Operative times were recorded.

## Results

**Fig. 5:**
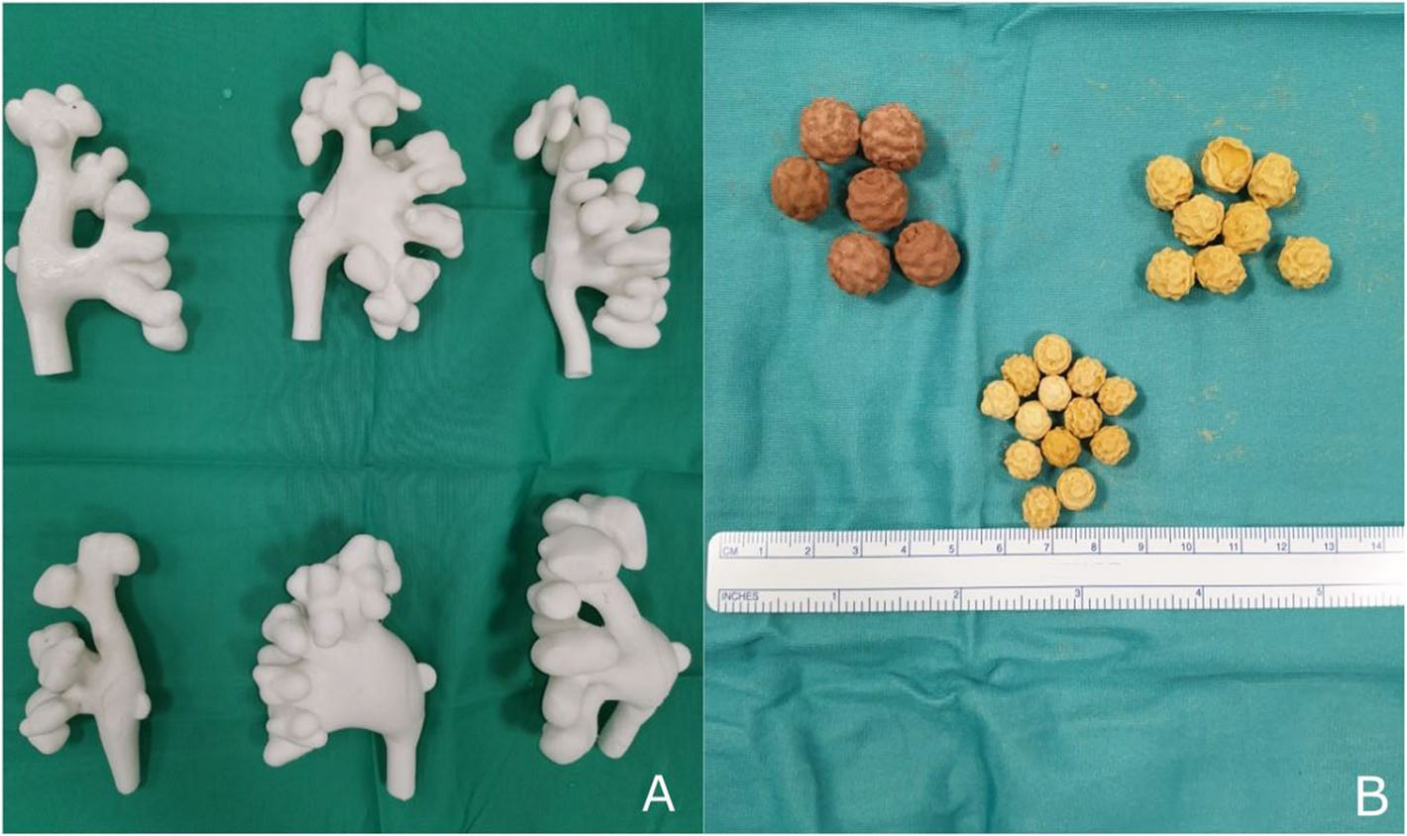
**A** 3D Printed training models of different pelvicalyceal systems. **B** Training stones in different sizes, brown = hard stones, yellow = soft stones

Six training models were obtained (Fig. 5A). Overall, cost totalled €200–400 per anatomical model, subdivided into: €70 of material costs, €80 of fixed depreciation costs for software and 3D printers, €50–250 of direct labour costs. The range in labour cost was determined mainly by the complexity of the anatomies, allowing that the dedicated bioengineer needed more working hours to digitally optimize more intricate segmented models. Three different moulds were obtained and stones with diameters of 6-8-10 mm were produced (Fig. 5B). Production cost per each mould was €300, including €50 of material costs and €250 of direct labour costs, stone manufacturing cost averaged €2 per stone.

**Fig. 6:**
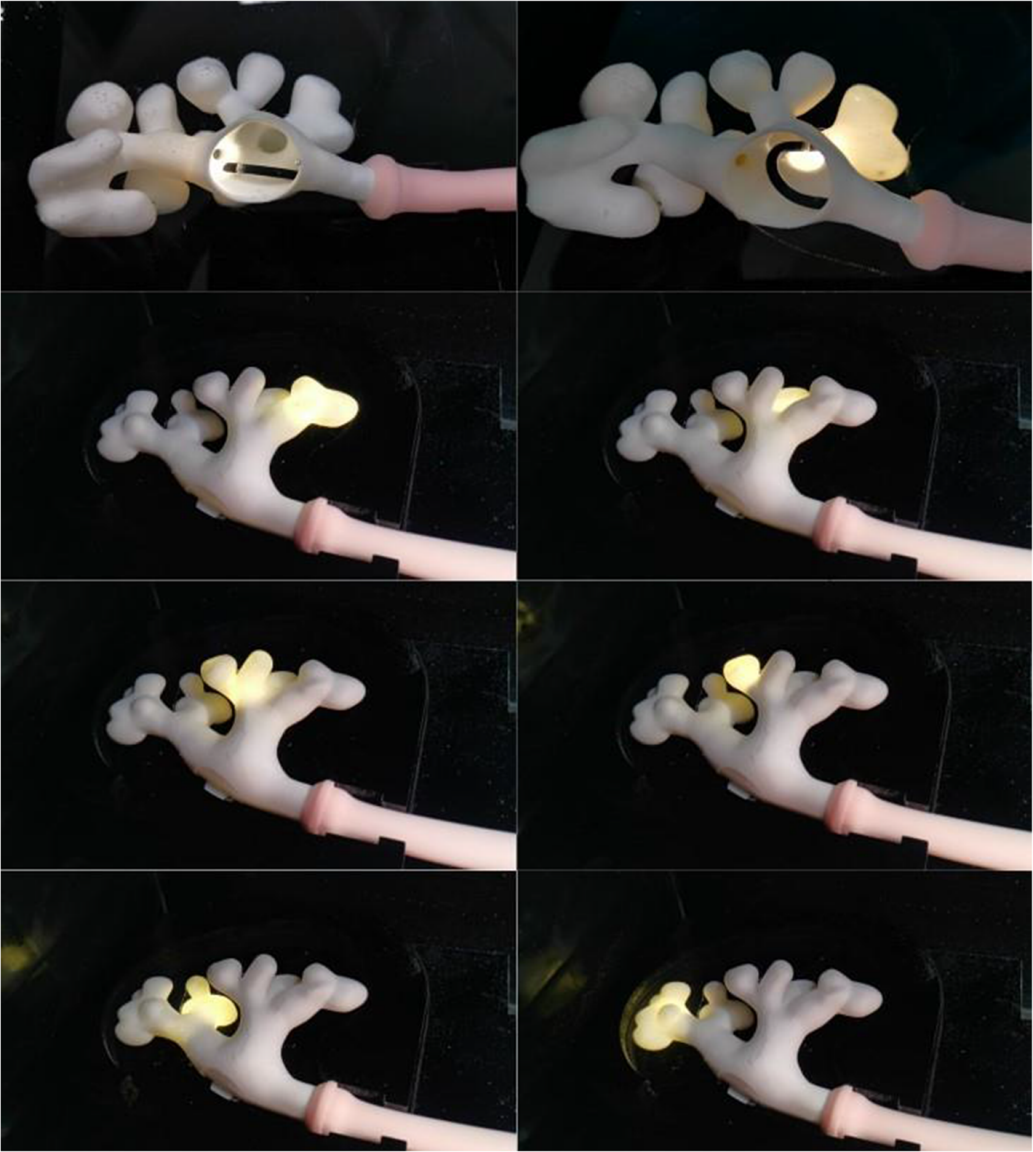
Navigation, the model allowed exploration of each single calyx using a flexible ureterorenoscope

The models showed an extraordinary anatomical accuracy and resemblance to the real upper urinary tract, it was possible to navigate the renal pelvis and explore each single calyx with both the ureterorenoscopes (Fig. 6). The complexity score was used to select a model for RIRS testing. Subjective assessment during navigation confirmed no significant friction between the models and the instruments.

**Fig. 7:**
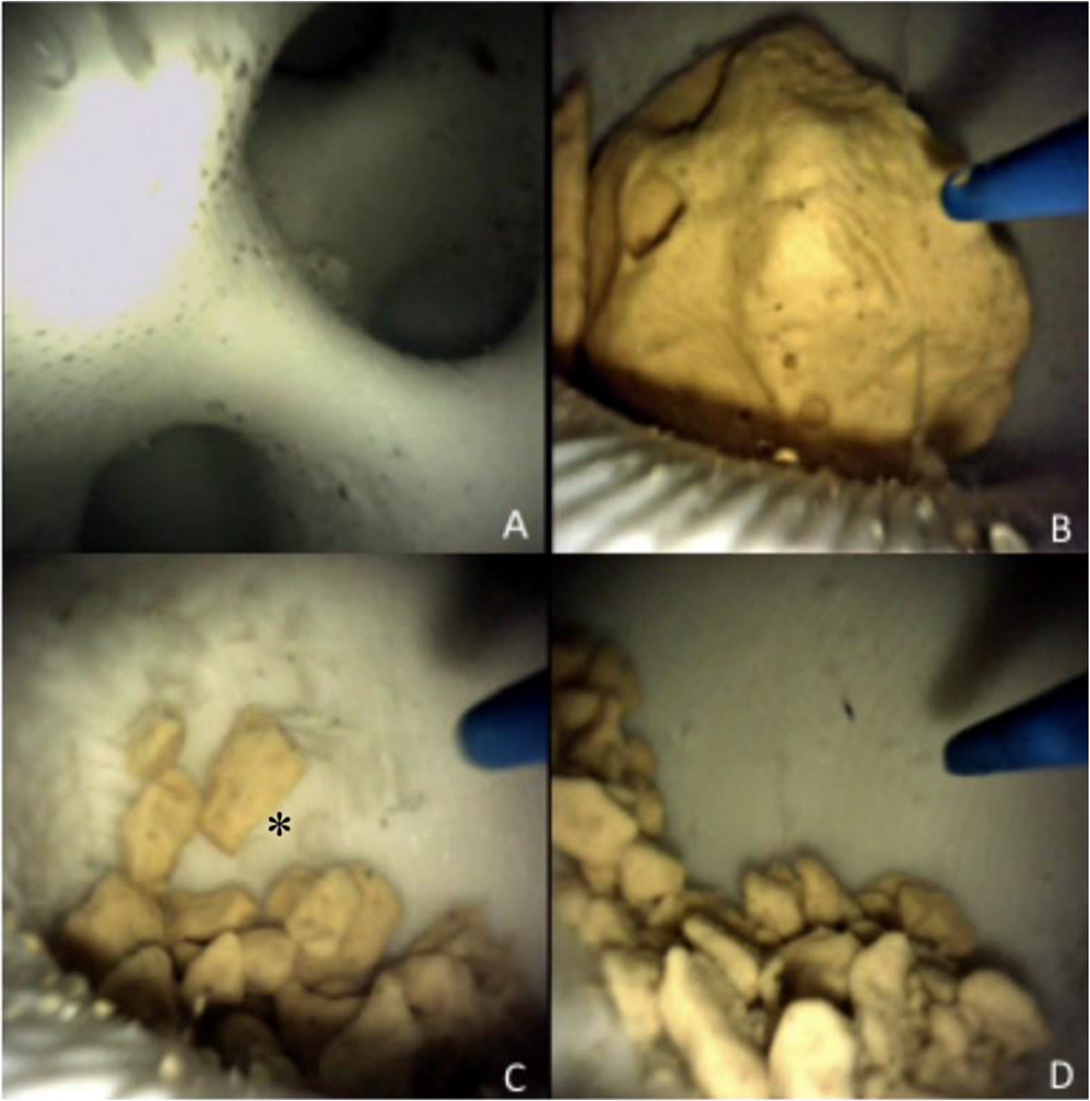
Endoscopic view during the simulation. **A** Flexible URS. **B** Identification of an 8 mm soft stone. **C** Laser stone fragmentation, small superficial tears of the model acting as a proxy of mucosal damage can be seen (*). **D** Stone debris after pop-corning

A medium complexity model was used for RIRS simulation. Five expert endourologists conducted several lithotripsy trials using both soft and hard stones of each available calibre. The stones were inserted in the water filled models through the dedicated hatch. The laser setting used during the simulation were: dusting (12 Hz, 0.6 J, 700 µs pulse width), fragmentation (5 Hz, 1.5 J, 350 µs pulse width), pop-corning (10 Hz, 1.0 J, 700 µs pulse width). Both types of stones reproduced real-life conditions in all of the three lithotripsy settings described (Fig. 7), with time needed to complete the RIRS being similar to elective cases. Stone relocation and fragment extraction were successfully performed. Operating times for the simulation of a RIRS of a 8 mm soft stone located in an inferior calyx are shown in Table 1. Stone debris removal at the end of the procedure proved to be easy, as the upper urinary tract model could be detached from the part-task trainer and washed under running water.

**Table 1:** Expert operating times for the RIRS of a lower calyx 8 mm soft stone using fragmentation setting, lithotripsy time includes stone relocation into an upper calyx. *SD* Standard Deviation

Expert	Navigation (sec)	Lithotripsy (sec)	Fragment Extraction (sec)	Simulation Duration (sec)
**A**	**92**	**592**	**200**	**884**
**B**	**82**	**668**	**174**	**924**
**C**	**70**	**673**	**123**	**866**
**D**	**130**	**561**	**200**	**891**
**E**	**101**	**605**	**153**	**859**
**Mean (SD)**	**95,0 (22,7)**	**619,8 (49,0)**	**170,0 (32,8)**	**884,8 (25,5)**

**Fig. 8:**
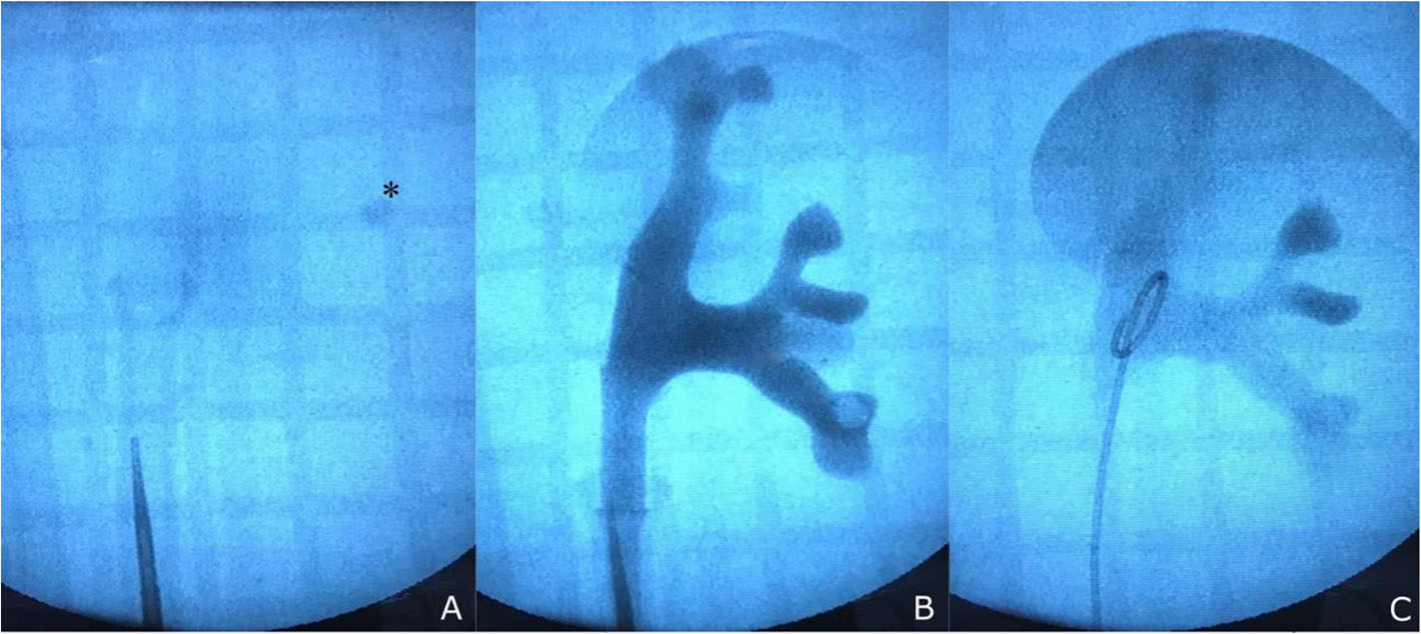
**A** Fluoroscopy, a radiopaque training stone is identified (*). **B** Retrograde pyelogram, the training stone can be observed in a lower calyx. **C** Fluoroscopic view after placement of a double-J stent

The simulation included fluoroscopy and retrograde pyelogram using iodinated contrast agent. The upper urinary tract models demonstrated to be radiotransparent while the simulation stones were radiopaque. The pyelogram showed exceptional resemblance to real intraoperative conditions. Guidewire placement in the upper pole calyceal system, access sheath removal and double-j ureteral stent placement were successfully performed under fluoroscopic guidance (Fig. 8). Minimal leakage of contrast medium from the pelvis hatch was seldom observed during high pressure RIRS. No leakage from the junction between the model and the ureter occurred. As the leakage was minimal, it never compromised the simulation of RIRS. If necessary, leaked contrast medium could be removed between simulations by draining the part-task trainer box and replacing the water.

One single model was employed for more than fifty RIRS simulations, without any impairment of its integrity due to repetitive strain or heat transfer from the laser fibre. Overall, the 3D printed model of upper urinary tract fitted in the part-task trainer allowed a high-fidelity complete simulation of a RIRS.

## Discussion

Currently available evidence defining the learning curve in RIRS and the case-load necessary to achieve proficiency is scarce and based on case-series only [11]. An exact identification of the learning curve also depends on the endpoints selected to certify proficiency, which might include: measures of operative outcomes, patient safety and task efficiency. Heterogeneity in reporting outcomes, patients’ differences in anatomy/stone burden and prior surgical expertise of the trainee with RIRS or other endourological techniques can hinder the exact definition of the learning curve [11]. Granted this, available studies identify a case-load of 50–60 cases to reach a plateau in terms of operative outcomes in RIRS [12–14], and 50–100 cases to reduce severe operative complications [12, 15]. Complying to a minimum case-load of 50 RIRS per trainee might represent a difficult goal for the training programs outside of the frameworks of dedicated endourology fellowships, therefore leading to the possibility of an inadequate or uneven level of expertise with the surgical technique at the end of the residency program.

Our model allows to train in each surgical step of RIRS. Its strengths are the high fidelity of the simulation, the usage of radiotransparent polymers for the upper urinary tract and radiopaque mixtures for the stones, the high variety in anatomical complexity, the several different options in stone size, shape or positioning and the virtually endless reusability. These features could enable the design of a standardized modular training program, tailored to the initial surgical expertise of each trainee, aimed at safely developing transferrable surgical skills which could reduce the case-load required to achieve proficiency.

Experimentation with the model identified four main differences with real life cases, which were recognised as possible limitations. Firstly, bleeding due to accidental mucosal damage during lithotripsy was not reproduced, it was however observed that unintended activation of the laser fiber against the model during lithotripsy produced superficial tears which were identified as a satisfying proxy for mucosal damage during training. As the amount of damage was only minimal, it never compromised reusability. Secondly, the model reproduces RIRS in a static environment, not accounting for the potential movement of the stone during lithotripsy due to the diaphragmatic excursions caused by pulmonary ventilation. This represents a drawback to the complete fidelity of the simulation which does not allow to fully reproduce one of the unique challenges associated with the procedure. Thirdly, as stones can be inserted in the model only through the dedicated hatch, it is not possible to position a stone wider than the calyceal infundibulum into a calyx, not allowing to simulate RIRS of a probable real-life scenario. Lastly, the Cook URS part-task trainer does not allow to change position, shape and size of the ureteric orifices or to introduce ureteric kinks, therefore impeding to control the level of complexity of the ureteric catheterization, guidewire placement and advancement of the access sheath.

Several other benchtop simulators for flexible URS have been recently presented. Villa et al. reported on a 10 days-long training program for flexible URS and stone relocation using a series of 4 low-fidelity benchtop models of the upper urinary tract called K-box (K-BOX®, Porgès-Coloplast, France) [16]. The model was targeted at novices in endourology and helped improving task efficiency, navigation and relocation skills. Another benchtop model was presented by Al-Jabir et al., the Advanced Scope Trainer (Mediskills, Northampton, UK) was a realistic tensile elastomeric silicone model of the whole urinary tract built to simulate every step of flexible URS and lithotripsy [17]. The model included also an enlarged kidney and a tortuous ureter to change the level of anatomical complexity. It was praised for its realism and educational value by medical students, trainees and experts over the course of multiple training sessions. The authors reported some degree of friction during the simulation and a high difficulty in ureteral orifice catheterization which required experts to assist during this phase of the procedure. A complete version of the Cook part-task trainer used in this study was described by Blankstein et al., the complete trainer included three different parts: a dual calyceal system, a left kidney-ureter and bladder system and a tortuous ureter [10]. Each individual part also included a suction pump for debris removal. The authors described a 2 weeks-long training program on guidewire placement, access sheath insertion, flexible URS and stone repositioning. A statistically significant improvement of performance metrics was observed among novices and junior trainees.

Out of the other benchtop simulators presented, none offers the degree of variation in anatomical complexity of the upper urinary tract achieved which the six different interchangeable models described in this study. Furthermore, none of these models allows to simulate patient’s breathing movements or mucosal bleeding during the training sessions. Studies involving trainees and experts to validate the complexity score, assess face, content and construct validity of our model and to objectively compare its value as a simulator with other described benchtop models are in progress. Such studies could also allow to design training programs for RIRS based on Objective Structured Assessment of Technical Skills (OSATS) [18], aiming to evaluate performance improvement after the training sessions.

Our production process proved to be feasible and reproducible, several more models could be printed aiming to build a full catalogue of different anatomies encompassing most of the real life variations encountered during RIRS. The stones could be modelled in different shapes to increase the complexity of the procedure and reproduce challenging scenarios such as RIRS of impacted or partial staghorn stones. Additional design post-processing of the pelvis hatch might lead to obtaining a completely watertight system. Manufacturing costs were low and comparable with other benchtop models also thanks to the outsourcing of the design and printing process to a dedicated bioengineering company. An operating theatre was required to experiment with our simulator due to safety protocols related to the use of holmium laser and ionizing radiations with fluoroscopy. Despite this, our model proved to be easily transportable, therefore a complete simulation of flexible URS and lithotripsy without fluoroscopy could also be arranged in an office-based scenario fitted with adequate eye protective equipment reducing the costs associated with the usage of an operating theatre. Low cost and high transportability represent an added-value benefit of our model, in view of the very high costs and low transportability of virtual reality endourology simulators [5, 19, 20] and the logistic/maintenance costs associated with animal [19] or cadaveric models [21].

## Conclusions

The implementation of 3D printing in medicine introduced several new possibilities to shift the paradigms of surgical training. While no currently available benchtop model is capable of fully simulate a real-life RIRS, this study presents a 3D printed, high-fidelity series of models of upper urinary tract and stones which allowed to simulate every surgical step of the procedure. Objective validation studies with trainees and expert could lead to the development of a complete training curriculum in RIRS aimed at improving surgical skills in a completely risk free-environment. This might achieve a reduction of the case-load required to reach surgical proficiency while ensuring high patient safety.

## Data Availability

All data generated or analysed during this study are included in this published article.

## Abbreviations

CTU: Computerized Tomography Urogram
DICOM: Digital Imaging and Communication in Medicine
HU: Hounsfield Unit
RIRS: Retrograde Intrarenal Surgery
ROI: Region of Interest
URS: Ureterorenoscopy
OSATS: Objective Structured Assessment of Technical Skills
